# Heavy Metal Toxicity Effects on Plants

**DOI:** 10.3390/toxics10120715

**Published:** 2022-11-23

**Authors:** Ilektra Sperdouli

**Affiliations:** Institute of Plant Breeding and Genetic Resources, HAO-Dimitra, Thermi, 570 01 Thessaloniki, Greece; ilektras@bio.auth.gr

Although heavy metals are naturally present in the soil, geologic and anthropogenic activities increase the concentration of these elements to amounts that are harmful to plants. Some of these activities include the mining and smelting of metals, the burning of fossil fuels, the use of fertilizers and pesticides in agriculture, and the production of batteries and other metal products in industries, sewage sludge, and municipal waste disposal. Growth reduction as a result of changes in physiological and biochemical processes in plants growing on heavy metal-polluted soils has been recorded. Some of the heavy metal phytotoxic manifestations include the disturbance of nutrient uptake and translocation, photosynthetic reduction (decrease in photosynthetic pigments, inhibition of electron transport, decrease in CO_2_ fixation, chloroplast disorganization, photooxidative damage), the generation of reactive oxygen species (ROS), the inhibition of antioxidative enzymes, cellular redox imbalance, DNA damage, and protein oxidation. The assessment of heavy metal toxicity’s effects on plants will enable the evaluation of heavy metal-tolerant plant species and their use for the phytoremediation of contaminated soils.

In the present work, the eleven papers published in this Special Issue are summarized, providing a picture of heavy metal toxicity’s effects on plants.

Two studies assessed the combined effects of zinc oxide nanoparticles (ZnO NPs) and bacteria on rice plants that were grown in water polluted with heavy metals (HMs). First, Akhtar et al., 2021 [1] showed that the combined treatments of ZnO NPs and bacteria play a significant role in plant tolerance level and the removal of HMs from water. Rice seeds primed with bacteria grown in HM-polluted water containing ZnO NPs (5 mg/L) showed a reduced uptake of HMs in roots, shoots and leaves, thus resulting in increased plant growth. Furthermore, their combined effects also reduced the bioaccumulation index and metallothionine (MTs) content and enhanced the tolerance index of plants. This study suggested that the synergistic treatment of bacteria with lower concentrations of ZnO NPs helped plants to reduce heavy metal toxicity, especially Pb and Cu, and enhanced plant growth.

Next, the study by Akthar et al., 2022 [2] explored how supplementation with lower concentrations of ZnO NPs interacting with bacteria may regulate the remediation mechanism at the proteomic and molecular levels of rice plants that are grown in HM-polluted water. The results revealed that the maximum nitrogen and protein content was observed in the roots, shoots, and leave of the plant grown using a combined bacteria–ZnO NP treatment under HM stress as compared with plants grown without or with individual treatments of ZnO NPs and bacteria. Plants that were grown in HM-polluted water have the highest expression levels of stress-induced genes such as myeloblastosis (*Myb*), zinc-finger protein (*Zat*-12), and ascorbate peroxidase (*Apx*), while the combined effect revealed minimum expression as compared with individual treatments. Overall, the findings of this study demonstrated that the combined effect of ZnO NPs and bacteria lowered the stress-induced gene expression, while it increased the nitrogen protein content and protein expression in plant grown under HM stress. 

As arsenic (As) contamination has emerged as a serious public health concern worldwide because of its accumulation and mobility through the food chain, the study by Khan et al., 2021 [3] planned to evaluate the effect of *Bacillus subtilis*-synthesized iron oxide nanoparticles (Fe_3_O_4_ NP) on rice growth under arsenic stress. The results revealed that a lower concentration of Fe_3_O_4_ NPs inhibits the arsenic level significantly and enhances plant growth, whereas a higher concentration fails to do so. It seemed that Fe_2_O_3_ NPs have different effects due to their dosage. Further research is needed to identify the basic intracellular mechanism of *Bacillus subtilis* to synthesize Fe_3_O_4_ NPs and subsequently increase their efficiency to remediate arsenic-contaminated water. This study suggests that *Bacillus subtilus*-synthesized iron oxide nanoparticles can be used as nano-adsorbents in reducing arsenic toxicity in rice plants and therefore, it is highly recommended to develop and subsequently commercialize them. 

Since co-cropping is an eco-friendly strategy to improve the phytoremediation capacity of plants growing in soils contaminated with heavy metals such as cadmium (Cd), the study by Wang et al., 2021 [4] aimed to characterize the effects of co-cropping Indian mustard (as an accumulator) and silage maize (as a main crop), and peat application on plant growth and Cd uptake, in order to evaluate the potential of this co-cropping system to phytoremediate a Cd-contaminated acid paddy soil. Taken together, the results demonstrated that silage maize should be co-cropped with Indian mustard at an appropriate density in Cd-polluted soils to achieve simultaneous remediation of Cd-contaminated soils (via Indian mustard) and the production of crops (here, silage maize). Peat application was shown to promote the removal of Cd from soil and the translocation of Cd into shoots and could contribute to enhanced phytoremediation of Cd-contaminated acid paddy soil.

Next, a study that evaluated the use of commercial charcoal and lime in lowering the concentration of Cd in cacao grown in tropical soil [5] is included in this Special Issue. The charcoal was characterized by microscopy and by adsorption tests, and it proved to be a material with macropores, with a maximum capacity of 8.06 mg Cd g^−1^ and favorable kinetic behavior. The results also showed that the application of liming decreased the mobility of Cd toward the seedlings, with the liming combined with charcoal leading to the absence of Cd in the cocoa seedlings. The findings, although limited to a small scale, demonstrated the possibility of applying low-cost and easy-to-handle amendments for the control of Cd in cocoa plantations.

Studies on Cd stress responses are available primarily in cereals, with minimal information reported in legumes [6]. Lentil (*Lens culinaris* Medik.) is an important legume crop cultivated worldwide as a source of dietary protein. This study evaluated the antioxidative and growth responses of two lentil varieties with differing seed Fe contents to Cd toxicity. It was revealed that Cd stress significantly affects lentil growth through oxidative damage. Cd stress tolerance in the biofortified genotype was attributed to strong antioxidant potential and improved growth in toxic environments due to the high seed Fe content. It is suggested that the biofortification of lentil varieties may help reduce malnutrition and improve Cd tolerance. 

The following paper included in this Special Issue evaluated the mitigative role of excess Zn^2+^ supply in Cd^2+^ uptake/translocation and toxicity in *Salvia sclarea* L. plants [7]. It was demonstrated that *S. sclarea* plants exposed to Cd^2+^ toxicity accumulated a significant amount of Cd^2+^ in their tissues, with higher concentrations in the roots than in the leaves. The accumulated Cd^2+^ led to a substantial decrease in photosystem II (PSII) photochemistry and disrupted the chloroplast ultrastructure, which coincided with an increased lipid peroxidation. Zinc application decreased Cd^2+^ uptake and translocation to leaves, while it mitigated oxidative stress, restoring chloroplast ultrastructure and ameliorated the adverse effects of Cd^2+^ on PSII photochemistry. It was concluded that excess Zn^2+^ application eliminated the adverse effects of Cd^2+^ toxicity, and therefore it could be used as an important method for low Cd^2+^-accumulating crops, limiting Cd^2+^ entry into the food chain.

The study by Song et al., 2022 [8] evaluated the alleviatory effects of silicon (Si) supplementation on ammonium (NH_4_^+^)-stressed salvia plants. Physiological disorders and typical NH_4_^+^ toxicity symptoms, as well as interrupted photosynthesis, were observed in the 100% NH_4_^+^-treated plants. Furthermore, cation uptake inhibition and oxidative damage were also imposed by the 100% NH_4_^+^ supply. In contrast, in the presence of Si, the NH_4_^+^ toxicity degree was attenuated and plant growth was ensured. Accordingly, the NH_4_^+^ toxicity appearance ratio decreased significantly. Furthermore, Si-treated plants showed an ameliorated photosynthetic ability, elevated internal K and Ca levels, and enhanced antioxidative capacity, as reflected by improved major antioxidant enzyme activities, as well as diminished accumulation of ROS (reactive oxygen species) and MDA (malondialdehyde). Those findings highlighted the agronomic importance of additional Si to nutrient solutions, especially pertaining to bedding plants at risk of NH_4_^+^ toxicity.

The aim of the following study was to investigate the ability of biochar amendment to reduce the availability of lead (Pb) in the soil and its uptake in lettuce (*Lactuca sativa* L. var. *adela*) [9]. Soils contaminated with Pb and amended with 5% biochar resulted in a ca. 50% reduction in the extractable (bioavailable) fraction of this toxic metal, limiting its accumulation in the leaves of lettuce grown in these soils by ca. 50%. A similar behavior was found for lettuce plants grown in a simplified soilless system, even with a much higher reduction (ca. 80%) and lower (1%) biochar addition. Increased cation exchange capacity and pH were likely the main factors limiting the bioavailability of Pb in the soil. Complexation with functional groups and precipitation/co-precipitation both on the biochar surface and in soil aggregates were likely the main mechanisms immobilizing this element. In conclusion, it is possible to suggest biochar as a useful and renewable biomaterial to allow the cultivation of lettuce in soils contaminated by Pb.

The article by Steliga and Kluk, 2021 [10] aimed to assess the possibility of using the *Melilotus officinalis* plant in the phytoremediation of soils contaminated with heavy metals (Zn, Pb, Cd) and coexisting petroleum hydrocarbons (TPH and PAH). Tests of the plant material showed that heavy metals taken up by *Melilotus officinalis* mainly accumulated in the root tissues, revealing a poor metal translocation from the roots to the shoots, which indicates that *M. officinalis* behaves as a phytostabilizer. Extensive toxicological monitoring, which is an element of novelty, carried out during the process of the biodegradation of petroleum pollutants (TPH and PAH) and phytoremediation with the use of *M. officinalis* allows one to track changes in toxicity that correlate with the reduction in pollutants (Zn, Pb, Cd, TPH and PAH) in the tested soils. The obtained research results confirm the correctness of the adopted concept of applying gradual soil treatment in biological processes (bioremediation and phytoremediation with the use of *Melilotus officinalis*) for the treatment of soil contaminated with heavy metals and coexisting petroleum hydrocarbons (TPH and PAH).

Finally, this Special Issue also includes a study that explored the tolerance of *Sasa argenteostriata* to zinc (Zn) stress and combined lead (Pb)–Zn stress [11]. The roots of *S. argenteostriata* were the primary organs accumulating Zn, and the absorption of Zn followed a trend of, when the concentration of Zn stress was low, the absorption of Zn by the roots increased with the increase in the concentration of Zn stress. Under combined Pb–Zn stress, the toxicity of heavy metals to *S. argenteostriata* was mainly caused by Pb. The roots were the main organs of *S. argenteostriata* that accumulated Pb. The presence of Zn could promote the absorption of Pb by the roots of *S. argenteostriata*, and the promotion was enhanced with increases in the Zn stress concentration. The results of this study can provide the basis for the application of *S. argenteostriata* in the remediation of the combined Pb–Zn contamination of soil. In the future, a long-term soil culture experiment should be conducted to further verify the toxicity of combined Pb–Zn stress on *S. argenteostriata* and the interaction between these elements.

In summary, the eleven manuscripts included in the present Special Issue demonstrated small steps in our continued understanding of the heavy metal toxicity effects on plants. This area of research is far from complete, and greater efforts would be needed from the scientific community to enable the assessment of heavy metal-tolerant plant species and their role for the phytoremediation of polluted soils.
